# Epi Info™: Now an Open-source application that continues a long and productive “life” through CDC support and funding

**Published:** 2009-04-30

**Authors:** Enrique Nieves, Jay Jones

**Affiliations:** 1 Centers for Disease Control and Prevention (CDC), Atlanta, USA

**Keywords:** Epi Info, Informatics

## Abstract

**About the authors:**

Enrique Nieves Jr is the Acting Director of the Division of Integrated Surveillance Systems and Services (DISSS), National Center for Public for Health Informatics (NCPHI), Coordinating Center for Health information and Service (CCHIS), Centers for Disease Control and Prevention (CDC), Atlanta, USA. Jay Jones is a BearingPoint Consultant to the CDC/NCPHI Division of Alliance Management and Consultation (DAMC), National Center for Public for Health Informatics (NCPHI), Coordinating Center for Health information and Service (CCHIS), Centers for Disease Control and Prevention (CDC), Atlanta, USA.

## Special feature

### Epi Info™ as an Open-source Application

The CDC’s National Center for Public Health Informatics (NCPHI) recently released the Epi Info™ code to the public, making it the first Public Health Information Network (PHIN) application to go open source. Because the code is freely available, other developers and other entities can work with the Epi Info™ code to add features, contribute enhancements, and make it a much better product. Going open source will also help Epi Info™ grow a wider user base, and as it grows, CDC hopes to attract more users who may want to help with development.

The Epi Info™ user base is already quite large, with over a million users worldwide. In its 22-year existence, many CDC developers and contractors have worked on Epi Info™, resulting in an amalgam of code that needed better organization and updating to bring it up to today’s standards plus to make it suitable for release to the Open-source community of developers.

The Epi Info™ code is comprised of many components, including Visual Basic, Access, SQL, C^#^, etc. The Open-source distribution; i.e., the Community Edition (CE) was released to the Open-source community with a MySQL plug-in and is posted at the Codeplex repository online. This build works with Ubuntu Linux, meaning that users are no longer required to use proprietary database applications such as Microsoft Access and MS SQL Server.

The Open-source release, coupled with the move to a CDC employee-based development team, may have contributed to recent rumors of the demise of Epi Info™. Epi Info™ is much alive, as evidenced by its recent teaming with InSTEDD to develop and test an adapter for data synchronization for Epi Info™. Mesh4x, an open-source application, synchronizes data between multiple databases. Even if the data resides on disparate systems, “Mesh4x can collect and distribute updates between two users over SMS, Internet (HTTP exchange), or through other available means” (Taha Kass-Hout, MD, MS, http://taha.instedd.org/2008/12/epi-info-and-mesh4x-prototype.html). With the successful testing of the Mesh4x module, CDC now recommends Mesh4x for anyone who needs to synchronize data using Epi Info™.

### Our Release Method	

To ensure our user base a successful transition to open source, there are currently three versions of Epi Info™. Epi Info™ 3.5.1 is the latest stable release of the application and has been deployed to the user base. Epi Info™ 7 and Epi Info™ Community Edition (CE) share identical code; however, Epi Info™ 7 is being developed at the CDC and Epi Info CE has been posted on the Codeplex website for Open-source developers to use.

- **Epi Info™ 3.5.1:** The current version is the final version to be released to its user base using the existing code. This version is not an Open-source product.
- **Epi Info™ 7:** This next version is currently in the early stage of the development process (Pre-Beta version). It will replace version 3.5.1 and although not an Open-source version, it will include modules and other enhancements created and/or suggested by the open source community, as well as by CDC developers.
- **Epi Info™ Community Edition (CE):** Epi Info™ 7 code that has been released to the open source community. Epi Info™ CE will be developed independently from Epi Info™ 7 by the open source community. Contributions from developers will be considered for inclusion in Epi Info 7™.

CDC created Epi Info™ CE for use outside the CDC firewall; new code developed by the Open-source community does not cross back over the firewall to CDC. The versions of Epi Info™ that are inside the CDC firewall (3.5.1. and Epi Info™ 7) have to meet strenuous federal security requirements. CDC placed Epi Info™ CE outside the CDC firewall to be able to receive contributions from developers throughout the world. Users who have access to Epi Info™ CE can develop and send CDC their contributions. CDC can then evaluate these contributions and determine if it is something that can be incorporated into Epi Info™ 7. Once a contribution is accepted, CDC will submit the build through certification and accreditation and other federal security requirements. These processes will ensure that CDC takes the best of what the open-source community offers while maintaining an internal application that meets federal security requirements for its Epi Info™ user base.

This process is not cyclical. CDC receives code enhancements from the open source community, but the inverse is not true. The Community Edition does not receive contributions from the CDC staff. This is necessary to ensure the open source application continues to be “free libre open source software” (FLOSS), and the CDC version meets federal security requirements and is vetted by the CDC before it goes to the user base within the agency. Eventually, there will be a fork in the two development efforts: Epi Info™ CE will be one product, and CDC’s Epi Info™ 7 will be a CDC-supported product that meets federal security requirements.

However during the entire process, the communication between users, developers, and sponsors will be ongoing and bi-directional. Currently, CDC is working on the communication process between users and the CDC Epi Info™ Team. CDC has a Web board for users to submit their enhancement ideas. There is also another board called MyEpiInfo, which is completely independent from CDC and provides a forum for developers to collaborate.

Though still very early in the process, CDC is currently working on a draft document that describes how Epi Info evolved into an open source product, which includes the collaborative work CDC did with their own Office of General Counsel in reviewing various open source licenses to consider the most appropriate license for CDC.

In the future, CDC envisions Epi Info™ as a suite of tools for epidemiologists. A set of very helpful tools for outbreak management and surveillance. The development of the software will be based on user feedback and contributions in an open source environment.

### Status Update

CDC’s Epi Info™ team is moving away from a contractor-based to a CDC employee-based development team, a transition that CDC has anticipated for many years. The CDC Team’s announcement that Epi Info™ code would be released to the open source community has been overshadowed by rumors that the application will no longer be supported. On the contrary, Epi Info™ continues to be funded by CDC and the team plans to release future versions based on feedback from users and the open source community. The current stable version (Epi Info™ version 3.5.1) is Vista compatible and is receiving great reviews. Although Epi Info™ 3.5.1 is not open source, the software remains free of charge.

## For more information

- Epi Info™ current website on CDC.gov: www.cdc.gov/epiinfo (available for download Epi Info™ 3.5.1)
- User Forum: http://cms.myepi.info/
- Epi Info™ Community Edition: http://www.codeplex.com/EpiInfo
- Epi Info™ Friends Group on Google: http://groups.google.com/group/EIFriends?hl=en (Restricted to invited members. To become a member, contact the group owner at andy.dean@gmail.com)
- Epi Info™ in Italy: http://www.epiinfo.it/
- Epi Info™ in Brazil (Portuguese): http://www.epiinfo.com.br/ead/
- Epi Info™ in Spain: http://www.cica.es/epiinfo/
- Mesh4X: http://code.google.com/p/mesh4x/

CDC/InSTEDD Collaboration blog: http://taha.instedd.org/2008/12/epi-info-and-mesh4x-prototype.html

